# Embodiment Comfort Levels During Motor Imagery Training Combined With Immersive Virtual Reality in a Spinal Cord Injury Patient

**DOI:** 10.3389/fnhum.2022.909112

**Published:** 2022-05-20

**Authors:** Carla Pais-Vieira, Pedro Gaspar, Demétrio Matos, Leonor Palminha Alves, Bárbara Moreira da Cruz, Maria João Azevedo, Miguel Gago, Tânia Poleri, André Perrotta, Miguel Pais-Vieira

**Affiliations:** ^1^Centro de Investigação Interdisciplinar em Saúde (CIIS), Instituto de Ciências da Saúde (ICS), Universidade Católica Portuguesa, Porto, Portugal; ^2^Centro de Investigação em Ciência e Tecnologia das Artes (CITAR), Universidade Católica Portuguesa, Porto, Portugal; ^3^ID+ (Instituto de Investigação em Design, Média e Cultura), Instituto Politécnico do Cávado e do Ave, Vila Frescainha, Portugal; ^4^Human Robotics Group, Centro de Sistemas Inteligentes do IDMEC - Instituto de Engenharia Mecânica, Instituto Superior Técnico, Universidade de Lisboa, Lisbon, Portugal; ^5^Serviço de Medicina Física e Reabilitação, Hospital Senhora da Oliveira, Guimarães, Portugal; ^6^Serviço de Neurologia, Hospital Senhora da Oliveira, Guimarães, Portugal; ^7^Plano de Ação para Apoio aos Deficientes Militares, Porto, Portugal; ^8^Centre for Informatics and Systems of the University of Coimbra (CISUC), Coimbra, Portugal; ^9^Institute of Biomedicine (iBiMED), Department of Medical Sciences, Universidade de Aveiro, Aveiro, Portugal

**Keywords:** embodiment/bodily experience, spinal cord injured (SCI), brain–machine (computer) interface, comfort and human perception, tactile, thermal, virtual reality

## Abstract

Brain–machine interfaces combining visual, auditory, and tactile feedback have been previously used to generate embodiment experiences during spinal cord injury (SCI) rehabilitation. It is not known if adding temperature to these modalities can result in discomfort with embodiment experiences. Here, comfort levels with the embodiment experiences were investigated in an intervention that required a chronic pain SCI patient to generate lower limb motor imagery commands in an immersive environment combining visual (virtual reality -VR), auditory, tactile, and thermal feedback. Assessments were made pre-/ post-, throughout the intervention (Weeks 0–5), and at 7 weeks follow up. Overall, high levels of embodiment in the adapted three-domain scale of embodiment were found throughout the sessions. No significant adverse effects of VR were reported. Although sessions induced only a modest reduction in pain levels, an overall reduction occurred in all pain scales (Faces, Intensity, and Verbal) at follow up. A high degree of comfort in the comfort scale for the thermal-tactile sleeve, in both the thermal and tactile feedback components of the sleeve was reported. This study supports the feasibility of combining multimodal stimulation involving visual (VR), auditory, tactile, and thermal feedback to generate embodiment experiences in neurorehabilitation programs.

## Introduction

Spinal cord injury (SCI) is often associated with impairment in motor, sensorial, vesical, gastrointestinal, and sexual functions (de Bruin et al., 1999; Hess and Hough, 2012; Ahuja et al., 2017; Scivoletto et al., 2018; Alizadeh et al., 2019; Holmes and Blanke, 2019) as well as a reduction in quality of life (Dijkers, 1997). In sensory processing, multiple changes in body representations have been reported, namely in interoceptive and exteroceptive domains (Lucci and Pazzaglia, 2015; Pazzaglia and Molinari, 2016; Leemhuis et al., 2019). Recent studies have demonstrated that rehabilitation programs involving brain–machine interfaces that use real-time decoding of neural activity, virtual reality (VR), exoskeletons, electrical stimulation, and tactile feedback; are useful for SCI rehabilitation not only because they allow replacing functions (e.g., walking), but also due to their ability to generate beneficial neuroplastic effects (Donati et al., 2016; He et al., 2018; Shokur et al., 2018; Benabid et al., 2019; Selfslagh et al., 2019). Namely, improvements in sensorial, motor, sexual, digestive, and urinary systems have been reported in SCI patients undergoing rehabilitation programs combining BMI training with VR, assisted gait training, and BMI controlled exoskeletons (Donati et al., 2016; Selfslagh et al., 2019).

The precise mechanism underlying these neuroplastic effects occurring in SCI neurorehabilitation programs involving embodiment and BMI controlled avatars/exoskeletons is still under investigation (Lenggenhager et al., 2013; Donati et al., 2016; Pozeg et al., 2017; Shokur et al., 2018; Leemhuis et al., 2019). It has been proposed that it may be associated with the sense of embodiment (i.e., experiencing the avatar’s as one’s own body) (Kilteni et al., 2012; Pazzaglia et al., 2018). Embodiment is characterized by the senses of ownership, agency, and self-location (Kilteni and Ehrsson, 2017) which reflect different aspects of interoceptive states of the body. For example, the different domains evaluated in an embodiment questionnaire (Peck and Gonzalez-Franco, 2021) are expected to have different neural mechanisms, and may be differentially affected in SCI patients (Leemhuis et al., 2019), therefore constituting potential targets for VR therapeutic use (Leemhuis et al., 2021a,b). The neural basis of embodiment involves multiple areas associated with processing of the human body or its parts, self-processing, as well as multisensory integration, namely the extra-striate area and the temporoparietal junction (Arzy et al., 2006), ventral premotor cortex, medial superior temporal area, and the Rolandic operculum (Bekrater-Bodmann et al., 2014; Braun et al., 2018). Therefore, embodiment seems to be dependent on the number and type of feedback received by the subject (Schettler et al., 2019) and is expected to produce positive responses in brain-body disconnection conditions, such as SCI, due to its potential to alter neuronal maps of body representations (Pazzaglia and Molinari, 2016; Leemhuis et al., 2019).

**FIGURE 1:**
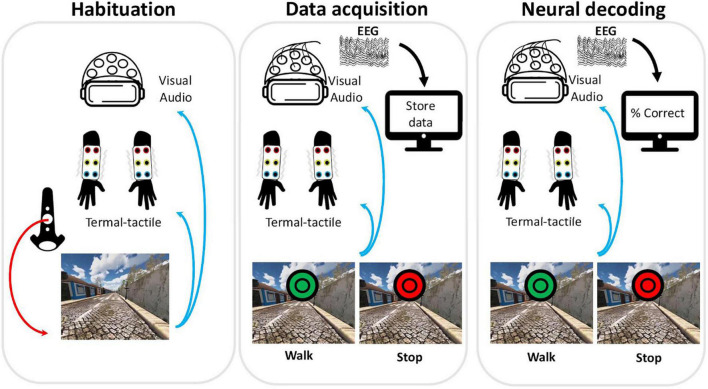
Setup and session phases. Each session consisted of three different phases: habituation, data acquisition, and real-time decoding. During habituation, the participant triggered each step of the avatar using the hand controller (red arrow) and received visual, auditory, and thermal-tactile feedback (blue arrows). During the data acquisition phase, neural data was recorded (black arrow) while the subject performed the motor imagery task and received visual, auditory and thermal-tactile feedback (blue arrows). In each session, a total of twenty Walk (green circles) and twenty Stop (red circles) cues appeared in the virtual reality scenario to indicate the type of motor imagery required in each trial. At each green (walk) cue the avatar would perform one step forward. The neural decoding phase was similar in all aspects to the data acquisition phase, with the exception that the classifier, trained with the data acquired in the second phase, would now decode in real-time neural activity recorded from the participant. In all phases the avatar moved independently of the neural activity. The subject received feedback of the neural decoding performance only at the end of the session.

Following in the footsteps of these previous rehabilitation programs and expanding the number of feedback modalities, we have developed a neurorehabilitation BMI setup including tactile, thermal, visual (VR), and auditory feedback (Figure 1). The general underlying hypothesis is that a more immersive (i.e., “realistic”) rehabilitation environment will lead to better performances in motor imagery tasks (Juliano et al., 2020) and consequently to increased or earlier neuroplastic effects (Donati et al., 2016; Vourvopoulos et al., 2019; Eng et al., 2020) in SCI patients. Such hypothesis is in line with previous proposals to use multimodal stimulation as an embodied approach to interact with the interoceptive and exteroceptive domains and consequently leading to neural plasticity (Pazzaglia and Molinari, 2016; Leemhuis et al., 2019).

It is not known, however, if increasing the number of feedback modalities will improve the overall effects of the rehabilitation program, or otherwise may become redundant or detrimental for BMI performance. For example, increasing the number of feedback modalities could lead to information overload (Ma et al., 2012) resulting in mental fatigue in the motor imagery task (Talukdar et al., 2019) or work as a distractor (Brandl and Blankertz, 2020), due to the excessive likeness of the avatar (i.e., uncanny valley effect) (Mori et al., 2012), or due to the additional paraphernalia worn by the patient. Also, the multimodal feedback could potentiate the effects of VR on the vestibular system, or the illusory lower limbs experience may lead to some unexpected form of discomfort (Konstantatos et al., 2009). In fact, while a significant effort has been made to guarantee the safety of the equipment used in these treatments [see He et al. (2018) for a review], little attention has been given to the levels of comfort associated with the embodiment experience. A description of the comfort levels experienced during embodiment in neurorehabilitation is therefore relevant since it may significantly condition the development and application of future protocols.

**FIGURE 2:**
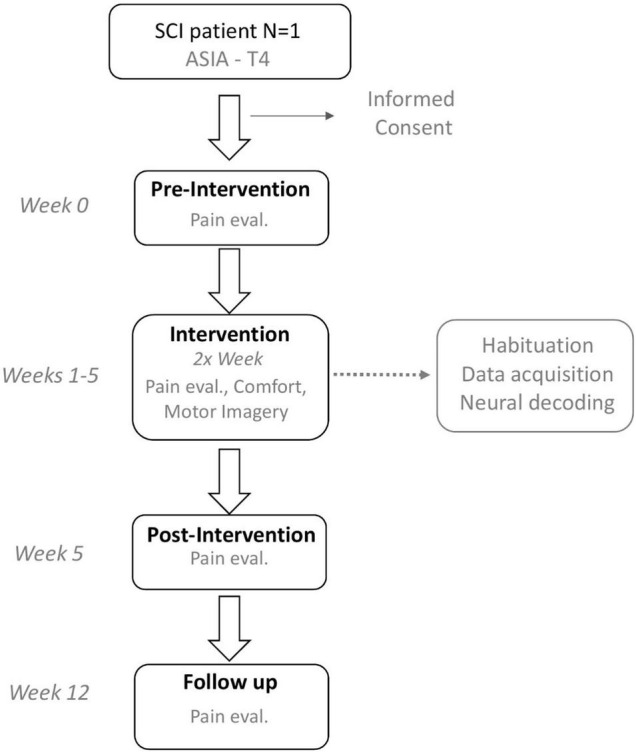
Study design. After an initial contact followed by informed consent a pre-intervention assessment was performed. In each session multidimensional assessment of comfort with embodiment experiences, VR side-effects, self-reported pain levels, and comfort with the use of the thermal-tactile sleeves was performed. Immediately after the last session, a post-intervention was performed (in the same day). Seven weeks after the post-intervention self-report pain levels were again evaluated.

**FIGURE 3:**
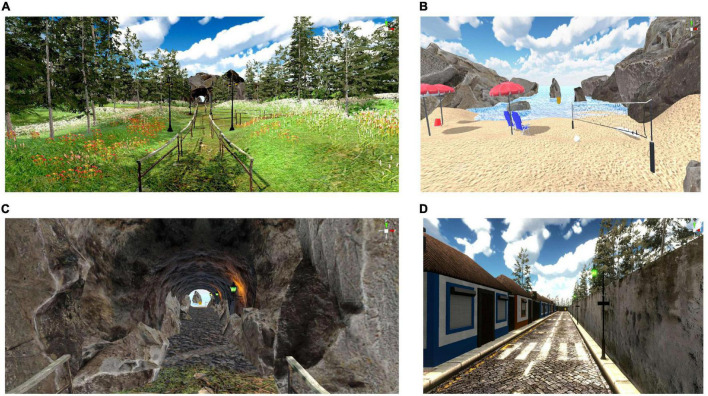
Examples of different scenarios used. **(A–C)** Scenario with sequential presentation of grass **(A)**, stone (inside a tunnel) **(B)**, followed by sand **(C)**, and water **(C)**. **(D)** Example of scenario with a single type of multimodal stimulation – Stone – in an urban environment (i.e., a street in the Azores, Portugal). For scenarios that involved a mix of elements, the multimodal feedback consisted of a mix of the different textures, temperatures, and sounds.

Here, we report the levels of comfort with embodiment of lower limbs experienced by a SCI patient tested in a multimodal feedback setup. The participant was an ASIA (Roberts et al., 2017) complete T4 SCI stabilized patient engaged in a rehabilitation program (Figure 2) involving the recording and analysis of neural activity, combined with the use of multimodal visual (VR), auditory, tactile, and thermal stimuli to create immersive scenarios (Figures 3A–D). The intervention used to assess comfort is the first part of a multiphase neurorehabilitation program described elsewhere (Donati et al., 2016). In the current phase, motor imagery content was focused on generating “Walk” versus “Stop” commands upon a visual cue appearing in the VR scenario. We expected that the embodiment experience of lower limbs, associated with the multimodal immersive environment, would not lead to significant or prolonged discomfort (with comfort considered as a score of five points or above in a seven point scale in all three domains of the adapted version of the avatar embodiment questionnaire: (a) perception of body qualities, (b) volitional control of movements, and (c) tactile perceptions) (Peck and Gonzalez-Franco, 2021), nor significant VR side effects [considered as a score above 2.0 in the 4-point Simulator Sickness Questionnaire (Kennedy et al., 1993)].

In addition, we also expected that the potential discomfort generated by the additional paraphernalia required to generate the thermal-tactile feedback, would be largely compensated by the quality of the immersive experiences allowed by the fully working setup, and therefore would not be described by the patient as a source of discomfort.

## Materials and Methods

### Subject and Timeline of Experiment

The study was approved by the CES- Hospital Senhora da Oliveira Ethics Committee (no. 15/2020). All experiments were performed in accordance with the Code of Ethics of the World Medical Association (Declaration of Helsinki) for experiments involving humans. The participating subject voluntarily filled an informed consent. The data presented here was collected between June and September 2021 at the Hospital Senhora da Oliveira, Portugal. The participant was a 52-year-old male with an ASIA complete T4 SCI stabilized (32 years) and a history of chronic low back pain post-lesion due to surgery (5 years). The individual results for each session are presented in Supplementary Informations 1–4. As presented in Figure 2, an initial baseline evaluation was performed followed by the intervention which lasted for a period of 5 weeks with two sessions per week (a total of 10 training sessions), followed by a final evaluation, and a follow up at 12 weeks.

### Apparatus

The system used here (Figure 1) consisted of a VR system composed of a headset with headphones (for visual and auditory stimuli) and two hand controllers (used during the habituation phase), (HTC Vive Pro Eye, New Taipei City, Taiwan), a pair of custom developed thermal-tactile sleeves capable of delivering different types of tactile and thermal stimulation (i.e., the feedback of the sole of the avatar’s feet was delivered to the forearms of the user) coupled to an external control system, allowing thermal stimulation in the temperature range of 18–35°C and complex tactile stimulation patterns through 6 independent vibration actuators in each arm, and EEG set up (16 channel, V-Amp, actiCAP; Brain Products GmbH, Gilching, Germany) with real-time recording and BMI decoding developed in OpenVibe.

Electroencephalography signals were acquired in real-time and confirmed through visual inspection of signals and by asking the user to open and close the eyes, to masticate, and to maintain eyes closed for a period of ∼10 s. During the acquisition and the neural decoding phases, an initial period of 20–30 s was used as baseline. An interval of 1 s after the appearance of the target was used for analysis. Neural data preprocessing and analysis was performed using existing the OpenVibe motor imagery with common spatial filter protocol (Renard et al., 2010). After the initial acquisition phase, data was preprocessed with a common spatial filter followed by training of 2-class linear discriminant classifier. The trained classifier was used during the neural decoding phase.

The software synchronizing and commanding the full apparatus was developed in Max (Cycling ’74, San Francisco, CA, United States), and the VR scenarios were developed in Unity (Unity Technologies, San Francisco, CA, United States). The details of the VR scenarios, as well as the controlling software and hardware used in this study will be described in detail in the VR environment section.

### Sessions

The intervention consisted of 10 sessions run twice a week with each session lasting between 70–90 min. This total time was divided in: (i) a period of 10 min for overall evaluation of comfort with the equipment as well as to gather information from the patient regarding the previous session, overall levels of pain and stress experienced at home or work, (ii) a period of 20–25 min where the subject interacted with the VR environment [this period was composed by three sequential phases: (a) habituation, (b) EEG baseline and neural data acquisition; and (c) real-time decoding; that are described in detail below], (iii) questionnaire application, and (iv) the remaining time corresponding to participant positioning, placing and calibrating the VR headset, confirming appropriate feedback from each different component of the multimodal feedback, checking EEG signal and impedance, as well as to remove the gear and cleaning the EEG gel. Sessions were run by 2–4 researchers to ensure that the full setup was working and to reduce the amount of time necessary to set the experiment. All comments related to the comfort of the embodiment experience, the apparatus, or the experiments organization; made by the participant were annotated and analyzed *a posteriori*.

### Comfort Evaluation

Comfort evaluation was performed at the end of each session, applying the adapted questionnaire (Peck and Gonzalez-Franco, 2021), the Faces pain scale (Gallagher et al., 2001), the Verbal pain intensity scale (Collins et al., 1997), the VAS Pain Scale (Bijur et al., 2003); and the Simulator Sickness Questionnaire (Kennedy et al., 1993). The overall goal was to evaluate comfort with the embodiment experiences of lower limb movement, while guaranteeing that no increases in pain would be present, or otherwise, VR side effects would occur. In addition, because we have developed a sleeve capable of delivering thermal and tactile feedback to the user, the participant was also asked about ease of placement, ease of use, thermal comfort, and tactile comfort of the sleeve; as well as overall perceived effects in the task (e.g., if the sleeves contributed to the immersiveness of the experience).

**FIGURE 4:**
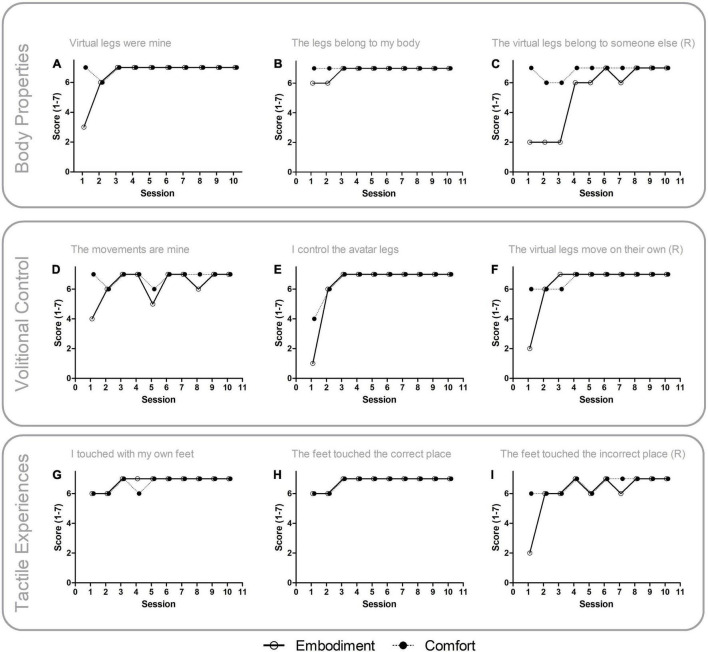
Embodiment experiences and comfort. Open circles indicate scores for the Avatar Embodiment Questionnaire items related to embodiment experience and filled circles indicate scores for items related to comfort of a particular embodiment experience. **(A–C)** Perception of body properties during embodiment experiences. **(D–F)** Volitional control during embodiment experiences. **(G–I)** Tactile perceptions during embodiment experiences. Note that the overall levels of comfort remained high even when, in the initial sessions, embodiment experience scores were low.

The avatar embodiment comfort questionnaire is an adaptation of the avatar embodiment questionnaire (Peck and Gonzalez-Franco, 2021) designed to evaluate the full range of experiences provided by our setup. The original questionnaire is composed by nine items set to evaluate three theoretical domains: perception of body qualities (three items), volitional control of movements (three items), and tactile perceptions (three items). In the present adaptation, we added to each original item, an additional question to evaluate the comfort of the experience. Therefore, in this adaptation each domain had three items related to the embodiment experiences (e.g., “I have felt the avatar legs as if they were my own legs”), which were now followed by three additional items evaluating the comfort of that experience (e.g., “How comfortable were you?” [with the experience of feeling the avatar legs as if they were your own legs]). The adapted questionnaire is presented in Supplementary Information 1. For analysis of the questionnaire data, the values from questions that were posed in the negative form were reversed. As an example, the question “The legs felt like they belonged to someone else,” which would have a value of 1 if the embodiment experience was maximum, was reversed such that this value now corresponded to 7 points. These specific questions are indicated with (R) in Figures 4C,F,I.

The participant was also allowed time (5–10 min per session) to freely describe any additional experiences that were considered as relevant (e.g., general concerns related to the general organization of the study, changes in pain at home, etc.).

Lastly, potentially relevant variables, such as extremes in external environment temperature or significant life-events (e.g., first day of work after sick-leave) were also recorded.

### Virtual Reality Environment

The use of VR was delivered through a setup that included VR headset with embedded headphones and two hand controllers. Altogether, a total of 16 different scenarios where available (i.e., including the initial habituation and training scenarios). These scenarios involved different types of landscapes, such that the participant could walk in one or more types of ground (grass, sand, stone, and water) in the same scenario. An example of the complexity of the VR scenarios used is presented in Figure 3. The participant was allowed to choose the scenario in each session, as well as the overall presentation of the avatar. This included choosing skin tone, clothes, shoes, etc., to increase immersiveness as well as the sense of control over the experimental environment for the participant. Even though the avatar was always in the first-person perspective, the movement of the hands (which were holding the VR hand controllers), or changes in head position, would allow the participant to observe parts of the avatar’s body during movement, more specifically, the arms and hands, lower part of torso, legs, and feet. In addition, one of the VR scenarios included a virtual mirror on the side of the track, such that the participant could see a virtual reflection of the movement of the avatar legs.

The movement animation of the avatar hands and body were developed using two distinct strategies: the hands, head and upper body movement was calculated in real-time using inverse kinematics (Buss, 2004) processed from the VR headset and joysticks position tracking and accelerometer sensors; the walking animation was developed as a pre-processed animation obtained from the recorded and motion capture analysis of a person using the ExoAtlet exoskeleton (walking, sit-to-stand, stand-to-sit) (Pais-Vieira et al., 2020). Therefore, the avatar walking motion simulates the motion of a user in an exoskeleton. This choice was made to prepare the patient for the more advanced phases of the training protocol (Donati et al., 2016) where an exoskeleton will be used.

### Interaction With Virtual Reality Environment

After having all the setup calibrated, the actual sessions started. The interaction with the VR consisted of three different phases: (a) habituation, (b) EEG baseline and neural data acquisition for classifier training; and (c) testing real-time decoding of neural activity without control of avatar (Figure 1). During the habituation period, the participant was asked to choose a scenario and to interact with it using the hand controllers that triggered the avatar steps. This period allowed identification of eventual adjustments that needed to be made to the sleeves, VR environment, or wheelchair position. At this point, the participant was also asked about potential side effects due to the interaction with the VR set up (Kennedy et al., 1993; Regan and Price, 1994). During the initial baseline period, 20 s of neural activity were recorded while the participant remained with the eyes open in the scenario to be used. This period established a clear separation between the initial period of preparation (i.e., checking multiple parameters, calibration, etc.) with the period of actual testing where it was necessary for the patient to concentrate and remain calm.

During the neural data acquisition phase, colored visual cues appeared, indicating “A trial is about to start” (gray cue; not shown in Figure 1), “Walk” (green cue), or think about “Stop” (red cue) (Figure 1). When a green cue appeared, the avatar performed a forward gait motion consisting of one step forward with a leg (alternating between left and right legs). From a total number of 40 trials in each session, 20 trials were associated with the cue to “Walk” and 20 trials with the cue “Stop.” At the beginning of this phase, the participant was instructed that, upon a green cue, he should try to imagine one of his legs rising and then stepping on the ground and in the following green trial he should imagine the same action for the other leg. Meanwhile, he was instructed that, when the visual cue was red, he should think about not moving and just stand in the same place (e.g., enjoying the scenario). After neural data acquisition, the common spatial filter and the classifier were trained, and the confusion matrix analyzed. If true positive values were above 70% for each category, training proceeded to the following phase. Otherwise, the neural data acquisition phase was repeated, and training proceeded.

In the last phase, neural decoding was performed in real-time using an adaptation of the OpenVibe é junto motor imagery protocol (Renard et al., 2010) communicating with the remaining setup. Here, motor imagery was used only to train the participant. He did not have control over the avatar, and he did not have immediate feedback on his performance. This means that, independently of the subject’s neural activity, the avatar would move when a green visual cue (Walk) appeared, and the avatar would remain still when the red (Stop) visual cue appeared. Although the next step of the rehabilitation protocol involves the actual control of the avatar using neural activity, in the present study we have specifically opted to run this intermediate phase without the brain control option. This was to allow evaluation of comfort levels during the experience of illusory lower limbs without the additional stress that could be generated by low performance during the real-time neural decoding feedback (i.e., the avatar not moving in a green cue trial). At the end of the session the subject was informed of the overall performance resulting from real-time neural decoding to allow feedback on the strategies that had been used in that session. Neural activity was decoded in real time, but no further offline analysis was performed.

### Statistical Analysis

Results are presented as mean ± standard deviation. Arbitrary units (a.u.) were used as units for the different questionnaires. An alpha value of 5% was considered significant for hypothesis testing. Comparison of variables with and without the use of the thermal-tactile sleeve was made with Mann–Whitney *U* tests. Data related to pain reports is presented linearly and in the same panel, although the different pain scales are evaluating different aspects and do not necessarily represent a linear scale (Myles et al., 1999), pain data relative to the three different scales is presented in the same graph solely for the purpose of presenting simultaneously the three measures for each session.

## Results

A total of 400 trials (200 “Walk” and 200 “Stop”) in 10 sessions, all of them using the VR setup, were acquired. Of these 10 sessions, 9 were performed with real-time decoding of neural activity. A total of 6 sessions were performed with the thermal-tactile sleeves and 4 sessions solely with the VR google and headphones. The raw values for the embodiment questionnaire, self-reported levels of pain, comfort with thermal-tactile sleeve, and performance in each session are presented in the Supplementary Information 1.

Analysis of the 7-point avatar embodiment comfort questionnaire revealed high levels of embodiment experiences in all three domains, namely in body properties (6.167 ± 1.621 a.u.), volitional control (6.333 ± 1.493 a.u.), and tactile sensations (6.567 ± 0.971 a.u.) (also see Supplementary Information 1 for embodiment values in each session). This adapted questionnaire also revealed that the participant was comfortable with the embodiment experiences in all three domains, namely: comfortable with body properties (6.9 ± 0.305 a.u.), comfortable with volitional control (6.7 ± 0.651 a.u.), and comfortable with tactile sensations (6.7 ± 0.466 a.u.). In Figures 4A–I, the details of the comfort with avatar embodiment questionnaire are presented. Each panel depicts, for all sessions, the results for each item related to the embodiment experience (empty circles) and the level of comfort of the participant with that experience (filled circles). Embodiment experiences were typically smaller in the first week (i.e., the first two sessions), and then increased throughout the remaining of the intervention. Meanwhile, the levels of comfort remained high throughout most of the sessions even when the embodiment experiences values were low, or the pain values were high (also see self-reported pain below). For example, in Figures 4A,C,E,F,I; which are associated with all three domains (each domain corresponds to one row comprising three panels), present large differences between the reported embodiment experiences and the overall level of comfort for those experiences, especially in the first sessions. In other words, the absence of embodiment experiences (which occurred only in the initial sessions), and the presence of embodiment experiences, were not associated with high levels of self-reported discomfort.

Analysis of VR side effects indicated few issues throughout the sessions (Table 1), with an overall score of 0.08125 ± 0.2741 a.u. in the 3-point Simulator Sickness Questionnaire. The presence of side-effects was more pronounced in the first session with 46.15% (6/13 side effects reported). The most frequent side effects were general difficulty focusing (3/13 = 23.08% of the effects reported), general discomfort and vertigo (both with the same frequency; 2/13 = 15.38% of the effects reported). Each one occurring in two different sessions. Lastly, during the interviews, the participant indicated that the side effects were temporary and restricted to the session period.

**TABLE 1:** Virtual reality side effects.

	Session number									
Side effect	1*	2	3	4	5	6*	7*	8*	9*	10*
General discomfort	1	0	0	0	1	0	0	0	0	0
Fatigue	0	0	0	0	0	0	0	0	0	1
Headache	0	0	0	0	0	0	0	0	0	0
Eye strain	0	0	0	0	0	0	0	0	0	0
Difficulty focusing	1	0	0	0	0	0	1	0	1	0
Salivation increasing	0	0	0	0	0	0	0	0	0	0
Sweating	0	0	0	0	0	0	0	0	0	0
Nausea	1	0	0	0	0	0	0	0	0	0
Difficulty concentrating	0	0	0	0	0	0	0	0	0	0
Fullness of the head	0	0	0	0	0	0	0	1	0	0
Blurred vision	1	0	0	0	0	0	0	0	0	0
Dizziness (eyes open)	1	0	0	0	0	0	0	0	0	0
Dizziness (eyes closed)	0	0	0	0	0	0	0	0	0	0
Vertigo	1	0	0	0	0	0	0	0	1	0
Stomach awareness	0	0	0	0	0	0	0	0	0	0
Burping	0	0	1	0	0	0	0	0	0	0
Total	6	0	1	0	1	0	1	1	2	1

*Sessions with thermal-tactile sleeve.

*Except for the first session, few VR side effects were observed throughout the sessions. The most common side effect was difficulty focusing. A 1 or a 0, respectively, indicate “slight” or “none” in a scale from 0 to 3 (none, slight, moderate, and severe).*

The participant typically chose complex scenarios involving a mixture of grass, rock, sand beach and water (such as in Figures 3A–C, where different parts of the same scenario are presented). When asked, the participant indicated that he had no preference for urban or natural scenarios. The participant’s favorite scenario included the avatar’s legs virtual reflection on the side of the walking path, allowing the patient to observe the gait motion while still observing the scenario through the first-person perspective.

**FIGURE 5:**
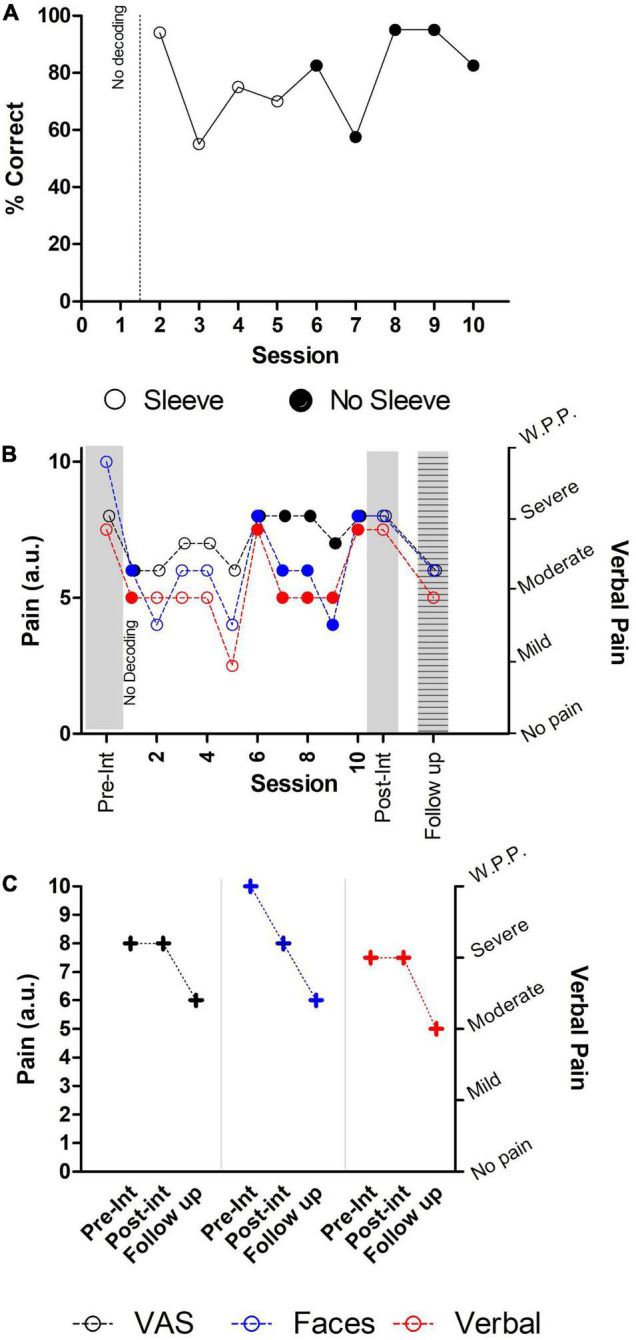
Performance, and self-reported pain levels. Filled circles indicate sessions were the thermal-tactile sleeve was used. **(A)** In the first session no neural decoding was performed. In the remaining sessions, performance varied, but was generally above chance. **(B)** Temporary decreases in pain were observed in some sessions, with post-intervention values showing a decrease solely for Faces pain scale. **(C)** Comparison between pre- and post-intervention with follow up at 7 weeks. An overall decrease was observed in all three self-reported pain scales. W.P.P. indicates Worst Possible Pain.

Neural decoding performance (Figure 5A) (also see Supplementary Information 2 for details of neural decoding in each session), collected from a total of 9 sessions, indicated that the participant could generate the neural commands associated with “Walk” and “Stop” with an overall performance of 75 ± 23% throughout the sessions. The performance in sessions with the use of the thermal-tactile sleeve was not above those without the sleeve (Sleeve: 82.50% ± 6.847; No Sleeve: 73.50% ± 8.407; Mann–Whitney *U* = 5.0, *P* = 0.2857, n.s.).

The results of the three different pain questionnaires applied during pre-intervention, the intervention sessions, the post-intervention evaluation and at 7 weeks follow up (Figures 5B,C) indicated that the intervention was associated with a reduction in the self-reported pain level of the Faces pain scale and a temporary reduction in the remaining scales (also see Supplementary Information 3 for details of self-reported pain in each session). In session 6, (a session where the thermal-tactile sleeve was used), a sudden increase in all three pain ratings was present. This session coincided with the first day of work after several weeks of sick leave for the participant and, in addition, with a particularly hot day (36°C).

Self-reports for the Faces pain scale (Figure 5B, blue circles) dropped from 10 points in the baseline evaluation, to an average of 5.8 ± 0.146 points throughout the intervention sessions, and then increasing to a final 8 points in the post-intervention evaluation. No differences were found between sessions with (filled circles) and without (empty circles) the thermal-tactile sleeve (Mann–Whitney *U* = 6.0, *P* = 0.3379, n.s.).

The VAS intensity of pain scale (Figure 5B, black circles) was 8 (in a 10-point scale) in the baseline evaluation, then dropped to an average of 7.1 ± 0.876 throughout the training sessions. However, during the post-intervention evaluation, self-reported intensity of pain again increased to 8 points. No differences were found between sessions with (filled circles) and without (empty circles) the thermal-tactile sleeve (Mann–Whitney *U* = 4.0, *P* = 0.1632, n.s.).

In the Verbal pain scale (Figure 5B, red circles), the participant described “severe pain” during the baseline, and in 7/10 = 70% of the remaining sessions indicated “moderate pain,” and in one session “mild pain” (1/10 = 10%). Lastly, the participant reported that, since he had started the intervention, he no longer felt like “cutting his trunk with a saw to end the pain.” In four sessions, where the thermal-tactile sleeve was used, pain ratings were described as moderate, and in two sessions as severe. Meanwhile, in three sessions without the thermal-tactile sleeve, pain was described as moderate and in one case as mild.

Comparison of self-reported pain at follow up, with the pre- and post-intervention scores, revealed an overall decrease in all three scales (Figure 5C). A two-point reduction was observed between pre-/post and follow up for the VAS intensity of pain scale (starting at 8, remaining in 8, and dropping to 6 a.u.). A four-point reduction was observed for the Faces pain scale (starting in 10, reducing to 8, and dropping to 6 a.u.); and a one level reduction in the Verbal pain scale (starting severe, remaining severe, and reducing to moderate).

Evaluation of the six sessions where the thermal-tactile sleeve was used, revealed an overall high level of satisfaction 6.75 ± 0.4423 a.u. (7-point scale) in the four-item scale (also see Supplementary Information 4 for details of thermal and tactile sleeve comfort levels in each session). Namely, items related to sleeve placement and sleeve usability were evaluated as 6.83 ± 0.4082 and 7.0 ± 0.00 a.u., respectively. Meanwhile items related to thermal comfort and tactile comfort were evaluated as 6.33 ± 0.5163 and 6.83 ± 0.4082 a.u., respectively. In addition, the participant indicated preferring sessions with the thermal-tactile sleeve because he would get a more precise notion of the length of the avatar’s steps due to the tactile feedback.

Analysis of the patient’s additional comments throughout the sessions suggested that the embodiment experiences had effects during the period spent in the laboratory as well as at home. Namely, the patient repeatedly described his experience of interacting with the setup as being positive, specifically through comments such as “there are not enough stars in the universe to rate how much I am enjoying this experience,” “this is the first time that I feel like I am standing up and walking in 30 years.” In addition, in one session, the patient reported having vivid dreams of performing sessions where he was walking with the avatar in an unspecified scenario.

In three sessions with scenarios involving water, the participant reported feeling his legs cold as soon as he entered the VR scenario. This was not exclusive of sessions with the thermal-tactile sleeve. This was considered as a pleasant experience by the participant. He reported being surprised but not uncomfortable, since he had never experienced cold feet or legs in the previous 30 years.

## Discussion

The present study describes high comfort levels with embodiment experiences in a SCI participant during a motor imagery task combining visual, auditory, tactile, and thermal feedback. These levels of comfort were high in all three domains of the embodiment experience (i.e., body properties, volitional control, and tactile experiences) with or without the use of the thermal-tactile sleeves. Even when the motor imagery scores were low, as occurred in the first sessions, comfort levels with the embodiment experiences remained high. Also, the physical apparatus itself, (consisting of two tethered thermal-tactile sleeves, one VR headset with headphones, and an EEG cap covered by an additional disposable plastic protection) did not induce significant discomfort during embodiment experiences, nor did it increase the VR side effects. Self-reported pain levels presented a modest reduction throughout the sessions, with a relevant decrease appearing at 7 weeks follow up. These findings, while limited in their scope and generalization, support the feasibility of testing visuo-auditory and thermal-tactile feedback in a SCI neurorehabilitation program.

Embodiment experiences are complex phenomena that involve multiple neural networks (Arzy et al., 2006; Kilteni et al., 2012; Kilteni and Ehrsson, 2017) and which may result in discomfort related to pain or to VR side effects (Li et al., 2011; Martini et al., 2013; D’Alonzo et al., 2019; Matamala-Gomez et al., 2019). In the present study, the participant reported high levels of comfort while interacting with the avatar and simultaneously receiving four different types of feedback. The results from the questionnaires, the observations made in the sessions, and the interviews performed; all indicate that the embodiment experiences started as soon as the participant interacted with the VR environment. These experiences were then intensified by the movements of the avatar (which resulted in multiple types of feedback indicating that the legs and the feet were moving properly and touching the ground as expected in normal gait) and by the transitions occurring in the scenario (e.g., moving from a part of the scenario associated with grass to another one with sand). Despite the participant’s reports that sessions with the full setup were more engaging than sessions without the thermal-tactile sleeve, no differences in accuracy of neural decoding, embodiment comfort levels, or self-reported pain ratings were found. On one hand, these results indicate that no detrimental effects in comfort or motor imagery performance were generated by the introduction of the thermal-tactile sleeve. On the other, as no clear improvement in performance, or permanent reduction in pain was observed when the thermal-tactile feedback was used; further studies with increased samples and an increased number of sessions, will be required to evaluate the true neurorehabilitation potential of the multimodal feedback setup used here (Ma et al., 2012; Donati et al., 2016; Pazzaglia et al., 2016; Talukdar et al., 2019; Brandl and Blankertz, 2020). Meanwhile, the overall reduction in the levels of pain, especially at follow up, and the patient’s reports of increased engagement in sessions where the thermal-tactile sleeves were used, support the use of this multimodal approach. Lastly, the comfort questionnaire used here consisted of an adaptation of a previous embodiment questionnaire (Peck and Gonzalez-Franco, 2021). Further uses of this questionnaire, and the inclusion of the comfort evaluation, will require a proper translation and cross-cultural validation to ensure that the original characteristics of the questionnaire are maintained.

Although the precise mechanism underlying modulation of the sense of agency in this study is not fully clear, our results suggest that interoceptive processing, namely through its effects in each different embodiment domain, may hold relevant clues. Detailed analysis of the body properties domain in the self-reported embodiment levels, revealed lower values during the first three sessions (Figure 5C) suggesting that, for this particular patient, multimodal stimulation took longer to have an effect in interoceptive states related to body properties. While most of the effects reported here are likely to be dependent on visual stimulation, it will be important to quantify separately, and in combination, the effects of each modality used, since the effects of multisensory modulation may differ for a particular embodiment domain. Such approach is in line with a previous proposal that, after massive neuronal lesions (such as occurs in SCI), multisensory stimulation may have the ability to reactivate neural pathways that became silent (Lucci and Pazzaglia, 2015).

We have opted to develop highly complex and realistic scenarios with the goal of increasing immersiveness, interest, and engagement to maximize comfort and improve performance in the task (Grangeon et al., 2012; Juliano et al., 2020). The combination of these scenarios and the multimodal feedback resulted in very few VR side effects (Kennedy et al., 1993; Regan and Price, 1994). We speculate that the cause for this is because we have limited the avatar movement to a single step in each trial. We have opted for this based on preliminary results obtained in a healthy subject that had previously reported severe and persistent nausea (>24 h) when the avatar moved several steps. In this healthy subject, reducing the number of steps revealed sufficient to significantly reduce VR side effects. It should be noted, however, that using a single step approach in the avatar movement may not be as rewarding as having the avatar taking multiple steps in each trial (as indicated by preliminary data from two healthy subjects who reported such an effect when testing the setup). These results suggest that future studies in SCI patients should consider a balance between one or multiple steps to maintain high levels of motivation (Thomschewski et al., 2017), while preventing VR side effects.

Another relevant result from the present scenarios is that the participant’s choices revealed an overall preference for scenarios with multiple types of feedback, namely for mixed scenarios with a sequence of grass-stone-sand-water. In future studies it will be important to combine psychological evaluation (Jeunet et al., 2015a) and EEG activity to identify which type of scenarios are better suited for each participant (Li et al., 2020; Vieira et al., 2021).

At the present time it is not known if the different types of feedback used here can lead to earlier or larger neuroplastic effects in SCI neurorehabilitation, when compared to the original neurorehabilitation protocol on which the present study was based (Donati et al., 2016). The limited set of data presented here, suggests that the ability to choose between different VR scenarios, combined with the multimodal feedback, have contributed to the participant’s overall motivation, possibly due to a combination of increased engagement and immersiveness (Jeunet et al., 2015b; Kerous et al., 2018; Lotte and Jeunet, 2018; Škola and Liarokapis, 2018; Škola et al., 2019; Huang et al., 2021), although the participant’s psychological profile may have also played a significant role (Jeunet et al., 2015a; Leeuwis et al., 2021).

Self-reported pain levels varied throughout the motor imagery sessions and were reduced in all three scales at follow up. These results suggest that the embodiment experiences generated during the task have the potential to induce a temporary reduction in self-reported pain scales and, in addition, support the notion that the use of the different combinations of scenarios or types of thermal-tactile stimulation did not increase baseline pain levels. Previous studies have reported temporary reductions in pain during training with VR scenarios in SCI (Villiger et al., 2013; Li et al., 2020; Austin and Siddall, 2021), multimodal stimulation in SCI (Pazzaglia et al., 2016), as well as in other clinical problems (Li et al., 2011; Pourmand et al., 2018) [however, also consider (Konstantatos et al., 2009) for an increase in pain]. Two recent reviews of studies using VR to treat neuropathic pain in SCI (Chi et al., 2019; Austin and Siddall, 2021) suggest that the use of VR can have immediate analgesic effects, but its long-term benefits still need to be investigated. One possible explanation for the reduction in self-reported pain levels in the present study, is that an analgesic effect may have occurred due to attention moving away from ongoing pain (i.e., distraction) (Bantick et al., 2002; Moseley, 2007; Malloy and Milling, 2010). However, such mechanism alone is unlikely to explain the reduction in pain reported by our patient at 5 weeks follow up. One potential alternative and/or complementary explanation to the distraction mechanism, is that the use of virtual reality may also be able to promote partial reorganization of the somatosensory pathways (Leemhuis et al., 2021b) and consequently modify neural activity related to deafferented and deefferented body maps during the sessions as well as follow up (Leemhuis et al., 2022). In addition, it will be important to determine the precise role that stimulating an unaffected zone (i.e., the forearm) may have in potentially boosting the sense of embodiment and agency in body parts with reduced access to sensorimotor information (Leemhuis et al., 2022). Changes in processing occur in affected and unaffected zones (Henderson et al., 2011), and therefore can be critical for embodiment as well as pain levels (Pazzaglia, 2022).

Our patient specifically reported that the use of the set up *per se* did not seem to change his ongoing pain, but instead that he could separate the ongoing pain from the actual embodiment experience, and the motor imagery task. Meanwhile, pain assessment in pre-/post-intervention and at 7 weeks follow up, revealed and overall reduction in all three scales. Future studies with an increased number of subjects will be critical to determine if self-reported pain reductions consistently occur, and whether patients are aware of them or otherwise, if some other variable may explain our present observations. Also, the present results indicate that it may be important to ask participants to keep a record of the frequency and intensity of pain episodes occurring at home. In addition to the values obtained in the self-reported pain scales, the participant also reported that overall pain levels, even prior to the intervention, were often correlated to the number and intensity of spasmodic lower limb activity at home (Priebe et al., 1996). Although we observed spasmodic activity in two different sessions, we could not identify any specific event that triggered such activity. Future studies with electrophysiological recordings (e.g., surface electromyography) may help determine if the spasmodic activity was related to the intervention (e.g., generating “Walk command”).

Performances in the motor imagery task, indicated that the participant could generate different neural activity patterns for the algorithm to decode “Walk” and “Stop” commands (Donati et al., 2016; Roosink et al., 2016; Selfslagh et al., 2019). These results indicate that delaying the performance feedback toward the end of the session did not preclude the participant from performing the task.

A small number of caveats should be considered in the present study. First, we have adapted a previous questionnaire by adding the question of “How comfortable were you with that experience?” to each question of the existing questionnaire. As this adaptation has not yet been properly validated and we have only analyzed one participant, the present results cannot be generalized. Second, we have not tested the full brain–machine interface setup here. Although neural activity was decoded in real-time, the movement of the avatar was not controlled by the participant’s activity. We have opted to perform the present study without the actual control of the avatar to ensure that the participant would not loose motivation due to the potential of low performances occurring, since this could bias responses related to comfort. We have meanwhile tested this patient in the setup with real-time control of the avatar and motor imagery performances were above chance. Lastly, it is important to note that the adapted Embodiment scale, the three pain scales, and the Simulator Sickness Questionnaire, are all self-reported scales. Therefore, a certain level of subjectivity and uncertainty must be considered when evaluating the present results. In future studies, it will be important to include additional physiological measures that can add other levels of evidence and reduce the weight of subjectivity.

## Conclusion

High levels of comfort with embodiment experiences were present when a SCI participant was tested for 5 weeks in a motor imagery task combining multimodal visual, auditory, tactile, and thermal feedback. This intervention resulted in a reduction of self-reported levels of pain at 7 weeks follow-up. The setup used did not induce significant discomfort nor generated significant VR side effects. These results support the feasibility of testing the effectiveness of neurorehabilitation programs based on embodiment through the application of a motor imagery task combining multimodal visual, auditory, tactile, and thermal feedback.

## Data Availability Statement

The original contributions presented in the study are included in the article/Supplementary Material, further inquiries can be directed to the corresponding author.

## Ethics Statement

The studies involving human participants were reviewed and approved by the Comissão de Ética para a Saúde- Hospital Senhora da Oliveira Ethics Committee (no. 15/2020). The participating subject voluntarily provided written informed consent to participate in this study.

## Author Contributions

AP developed the overall setup. AP and PG developed the virtual reality setup. AP, DM, and LA developed the thermal-tactile sleeves. CP-V, DM, TP, AP, MA, MG, and MP-V collected the data. CP-V and MP-V analyzed the data. All authors participated in the manuscript writing and agreed to the final version of the manuscript.

## Conflict of Interest

The authors declare that the research was conducted in the absence of any commercial or financial relationships that could be construed as a potential conflict of interest.

## Publisher’s Note

All claims expressed in this article are solely those of the authors and do not necessarily represent those of their affiliated organizations, or those of the publisher, the editors and the reviewers. Any product that may be evaluated in this article, or claim that may be made by its manufacturer, is not guaranteed or endorsed by the publisher.
