# Supplementary material for: Embodiment Comfort Levels During Motor Imagery Training Combined With Immersive Virtual Reality in a Spinal Cord Injury Patient
Source: Front Hum Neurosci. 2022 May 20;16:909112. doi: 10.3389/fnhum.2022.909112 (PMC9163805; doi:10.3389/fnhum.2022.909112)
Supplement: Supplementary file 1 [file Data_Sheet_1.PDF]

Supplementary Material

1 Supplementary Data

Supplementary Information S1 - Details of Embodiment questionnaire response

Questionnaire responses

Body properties

	Q1		Q2		Q3	
Session	Pic1	Comf1	Pic1	Comf1	Pic1	Comf1
**1**	3	7	2	7	6	7
**2**	6	6	2	6	6	7
**3**	7	7	2	6	7	7
**4**	7	7	6	7	7	7
**5**	7	7	6	7	7	7
**6**	7	7	7	7	7	7
**7**	7	7	6	7	7	7
**8**	7	7	7	7	7	7
**9**	7	7	7	7	7	7
**10**	7	7	7	7	7	7

Qpic 1. I have felt the avatar legs as if they were my own legs.

Qcomf.1How comfortable were you with that experience?

Qpic2. I have felt that the avatar legs were someone else's legs.

Qcomf.2 How comfortable were you with that experience?

Qpic3. I have felt as if the avatar legs were part of my body.

Qcomf.1How comfortable were you with that experience?

Volition control

	Q4		Q5		Q6	
Session	Pic1	Comf1	Pic1	Comf1	Pic1	Comf1
**1**	4	7	1	4	2	6
**2**	6	6	6	6	6	6
**3**	7	7	7	7	7	6
**5**	5	6	7	7	7	7
**6**	7	7	7	7	7	7
**7**	7	7	7	7	7	7
**8**	6	7	7	7	7	7
**9**	7	7	7	7	7	7
**10**	7	7	7	7	7	7

Qpic4. I have felt that the movements of the avatar legs as if they were my own leg movements.

Qcomf.1How comfortable were you with that experience?

Qpic5. I have felt that I could control the avatar legs as if they were my own legs.

Qcomf.1How comfortable were you with that experience?

Qpic6. I have felt that the avatar legs were moving on their own.

Qcomf.1How comfortable were you with that experience?

Tactile experiences

	Q7		Q8		Q9	
Session	Pic1	Comf1	Pic1	Comf1	Pic1	Comf1
**1**	6	6	6	6	2	6
**2**	6	6	6	6	6	6
**3**	7	7	7	7	6	6
**4**	7	6	7	7	7	7
**5**	7	7	7	7	6	6
**6**	7	7	7	7	7	7
**7**	7	7	7	7	6	7
**8**	7	7	7	7	7	7
**9**	7	7	7	7	7	7
**10**	7	7	7	7	7	7

Qpic7. When the avatar feet touched the ground, it seemed that I was touching the ground with my own feet.

Qcomf.1How comfortable were you with that experience?

Qpic8. It seemed as I felt like my feet were touched in the location I saw the avatar leg touched

Qcomf.1How comfortable were you with that experience?

Qpic9. It seemed as if the touch I felt was located somewhere between my own feet and the virtual feet.

Qcomf.1How comfortable were you with that experience?

Supplementary Information S2 - Details of neural decoding in each session

Session Performance

Session	% Correct
1	no decoding
2	94
3	55
4	75
5	70
6	82.5
7	57.5
8	95
9	95
10	82.5

Supplementary Information S3 - Details of self-reported pain levels in each session

Pain levels

Session	VAS	Faces	Verbal
pre-intervention.	8	10	Intense
1	6	6	Moderate
2	6	4	Moderate
3	7	6	Moderate
4	7	6	Moderate
5	6	4	Light
6	8	8	Intense
7	8	6	Moderate
8	8	6	Moderate
9	7	4	Moderate
10	8	8	Intense
post-intervention	8	8	Intense
follow up	6	6	Moderate

Supplementary Information S4 - Details of self-reported thermal and tactile sleeve comfort levels in each session

Tactile sleeve comfort levels

Session	placement	use	thermal comfort	tactile comfort
1	6	7	6	7
2	n.a.	n.a.	n.a.	n.a.
3	n.a.	n.a.	n.a.	n.a.
4	n.a.	n.a.	n.a.	n.a.
5	n.a.	n.a.	n.a.	n.a.
6	7	7	6	7
7	7	7	6	6
8	7	7	7	7
9	7	7	7	7
10	7	7	6	7

n.a. - non applicable
